# Hospital prescribing patterns of antibiotics in Zambia using the WHO prescribing indicators post-COVID-19 pandemic: findings and implications

**DOI:** 10.1093/jacamr/dlae023

**Published:** 2024-02-22

**Authors:** Steward Mudenda, Robert Chilimboyi, Scott Kaba Matafwali, Victor Daka, Ruth Lindizyani Mfune, Loriane Arielle Mobou Kemgne, Flavien Nsoni Bumbangi, Jimmy Hangoma, Billy Chabalenge, Larry Mweetwa, Brian Godman

**Affiliations:** Department of Pharmacy, School of Health Sciences, University of Zambia, P.O. Box 50110, Lusaka, Zambia; Department of Pharmacy, School of Health Sciences, University of Zambia, P.O. Box 50110, Lusaka, Zambia; Department of Pharmacy, Saint Francis’ Hospital, Private Bag 11, Katete, Zambia; Clinical Research Department, Faculty of Infectious and Tropical Diseases, London School of Hygiene & Tropical Medicine, Keppel Street, London WC1E 7HT, UK; Department of Public Health, Michael Chilufya Sata School of Medicine, Copperbelt University, P.O. Box 71191, Ndola, Zambia; Department of Public Health, Michael Chilufya Sata School of Medicine, Copperbelt University, P.O. Box 71191, Ndola, Zambia; Faculty of Health Sciences of Cotonou, University of Abomey-Calavi, Cotonou, Benin; Department of Medicine and Clinical Sciences, School of Medicine, Eden University, P.O. Box 30226, Lusaka, Zambia; Department of Pharmacy, School of Health Sciences, Levy Mwanawasa Medical University, Lusaka, Zambia; Department of Medicines Control, Zambia Medicines Regulatory Authority, P.O. Box 31890, Lusaka, Zambia; Department of Science and Technology, Ministry of Technology and Science, Maxwell House, Los Angeles Boulevard, P. O. Box 50464, Lusaka, Zambia; Department of Public Health Pharmacy and Management, School of Pharmacy, Sefako Makgatho Health Sciences University, Ga-Rankuwa 0208, South Africa; Strathclyde Institute of Pharmacy and Biomedical Sciences, University of Strathclyde, Glasgow G4 0RE, UK

## Abstract

**Background:**

Antimicrobial resistance (AMR) is a global public health problem that is fuelled by the inappropriate prescribing of antibiotics, especially those from the ‘watch’ and ‘reserve’ antibiotic lists. The irrational prescribing of antibiotics is particularly prevalent in developing countries, including Zambia. Consequently, there is a need to better understand prescribing patterns across sectors in Zambia as a basis for future interventions. This study evaluated the prescribing patterns of antibiotics using the WHO prescribing indicators alongside the ‘access, watch and reserve’ (AWaRe) classification system post-COVID pandemic at a faith-based hospital in Zambia.

**Methods:**

A cross-sectional study was conducted from August 2023 to October 2023 involving the review of medical records at St. Francis’ Mission Hospital in Zambia. A WHO-validated tool was used to evaluate antibiotic prescribing patterns alongside the AWaRe classification tool.

**Results:**

Out of 800 medical records reviewed, 2003 medicines were prescribed. Each patient received an average of 2.5 medicines per prescription. Antibiotics were prescribed in 72.3% of encounters, of which 28.4% were injectable. The most frequently prescribed antibiotics were amoxicillin (23.4%—access), metronidazole (17.1%—access), ciprofloxacin (8%—watch) and ceftriaxone (7.4%—watch), with 77.1% overall from the ‘access’ list. Encouragingly, 96.5% of the medicines were prescribed by their generic names and 98% were from the Zambia Essential Medicines List.

**Conclusions:**

There were high rates of antibiotic prescribing, including injectable antibiotics, which needs addressing going forward. It is crucial to implement targeted measures, including antimicrobial stewardship programmes, to improve future antibiotic prescribing in Zambia and reduce the risk of AMR.

## Introduction

Antibiotics are a class of medicines used to treat bacterial infections,^1,2^ and their discovery has revolutionized modern medicine to help save countless lives of patients suffering from infectious diseases.^2,3^ These medicines represent the most frequently prescribed therapeutic agents in medical facilities, especially in developing countries, where their usage has risen in recent years.^4–6^ However, their misuse and overuse, especially for self-limiting conditions such as upper respiratory tract infections (URTIs), have contributed to the development of antimicrobial resistance (AMR) worldwide.^6–11^ AMR arises when pathogenic bacteria undergo genetic changes that lead to a decrease or complete loss of susceptibility to antibiotics.^12^ AMR is an increasing concern as it is resulting in appreciable morbidity, mortality and costs, with costs rising as a result of extended hospital stays.^9,10,13–17^ Overall, it was estimated that in 2019, more than 1.27 million deaths globally were directly attributable to bacterial AMR, with potentially up to 4.95 million deaths globally associated with bacterial AMR.^10^ The morbidity and mortality associated with AMR will grow unless addressed, resulting in AMR increasingly being seen as the next pandemic.^18^ The greatest burden of infectious diseases and AMR is currently in sub-Saharan Africa.^10,19–22^

The increasing concerns about the rising rates of AMR, with their impact on mortality and costs, have resulted in a range of global and national initiatives including the development of a Global Action Plan (GAP) on AMR by the WHO to reduce AMR.^23–25^ The GAP has translated into national action plans (NAPs), with these plans at various stages of development and implementation across Africa.^25–31^ Alongside the instigation and development of the GAP and NAPs, the WHO also introduced the ‘access, watch and reserve’ (AWaRe) classification of antibiotics and associated guidance, which builds on the Essential Medicines List (EML) concept.^32–37^ The intention is to reduce the overprescribing of antibiotics, especially in ambulatory care, as well as reduce the prescribing of ‘watch’ and ‘reserve’ antibiotics, with their greater resistance potential.^6,32,37–41^ The AWaRe classification of antibiotics was developed in 2017 by the WHO to promote the rational prescribing and use of antibiotics, thereby reducing AMR.^42,43^ The AWaRe classification system has three categories of antibiotics including the ‘access’ (narrow-spectrum antibiotics with fewer side effects and lower risks for selection of AMR, hence recommended for empirical treatment of infections), ‘watch’ (recommended for use in hospital settings for sicker patients due to higher risks for selection of AMR) and ‘reserve’ (last-resort antibiotics reserved to treated infections caused by MDR pathogens).^32,33^

Before the development of the GAP on AMR, and subsequently NAPs, as well as the WHO AWaRe list and guidance, the WHO developed and implemented a range of prescribing indicators to promote a more rational use of antibiotics and other medicines in ambulatory care.^4,44^ The WHO prescribing indicators included an average number of medicines per encounter, the percentage of medicines prescribed by generic name, the percentage of encounters with an antibiotic prescribed, the percentage of encounters with an injection prescribed, and the percentage of medicines prescribed from the EML or formulary.^4,44,45^ The indicators provided a framework for evaluating prescribing practices, measuring antibiotic use, identifying potential areas for improvement, and monitoring changes over time.^44,46,47^ However, there have been concerns about whether these indicators fully measure the quality of prescribing in ambulatory care.^45^ This includes concerns with the actual quality of antibiotic prescribing for different infectious disease areas and not just the quantity prescribed, leading to recent initiatives, including the AWaRe classification of antibiotics and the AWaRe guidance by the WHO to reduce inappropriate prescribing of antibiotics and AMR.^32,33,37,38^ The initial target is that at least 60% of antibiotics prescribed must be from the ‘access’ list.^6,33,35,37^

There are concerns with the appropriateness of the current prescribing of antibiotics among hospitals across Africa, including hospitals in Zambia and other African countries, with typically high rates of empirical prescribing of antibiotics, especially from the ‘watch’ group.^39,40,48,49^ This is not helped by concerns of a lack of knowledge regarding antibiotics, AMR and antimicrobial stewardship programmes (ASPs), as well as the implementation of ASPs among healthcare professionals (HCPs) in some hospitals across Africa to improve future use.^24,50–53^ ASPs have been known to improve antibiotic prescribing in hospitals.^54–56^ These issues have been exacerbated by the lack of available personnel and resources to implement ASPs in low- and-middle-income countries (LMICs), including among African countries.^57^ This is now changing, with ASPs being routinely instigated across hospitals in Africa.^24,58–61^ We are also now seeing improved knowledge regarding antibiotics and AMR among HCPs in Africa, including Zambia, although more needs to be done.^62,63^

In Zambia, there are also issues and concerns with high rates of antibiotic prescribing in ambulatory care, with repeated high rates of prescribing of ‘watch’ antibiotics.^64,65^ This situation needs to be monitored and addressed as ambulatory care can account for up to 95% of antibiotic use in humans in LMICs.^66^ Consequently, this situation concerning antibiotic prescribing in ambulatory care has likely worsened post-COVID-19.^67–70^ This is because there has been appreciable prescribing of antibiotics for patients with suspected or actual COVID-19 across LMICs, despite limited evidence of secondary bacterial infections or coinfections.^67,71–78^ This also needs to be addressed going forward as part of the NAPs across Africa, including Zambia, to reduce AMR.

Additionally, studies have reported some deviations from treatment guidelines when antibiotics have been prescribed among healthcare facilities in Zambia.^39,48,64,74,79^ This is a concern, with adherence to guidelines increasingly seen as providing good-quality care and included in ASPs.^24,80–86^ Given this, it is important to assess the prescribing patterns of antibiotics in healthcare facilities across Zambia to identify areas for improvement and subsequently develop and implement appropriate strategies to promote their rational use as part of the ongoing NAP.^87,88^ Consequently, there is a paucity of information in Zambia concerning the prescribing patterns of antibiotics using the WHO prescribing indicators post-COVID-19 pandemic. Therefore, this study aimed to address this information gap by evaluating the antibiotic prescribing patterns in Zambia using the WHO prescribing indicators and the more recent prescribing targets based on the AWaRe classification and guidance post-COVID-19 pandemic.

## Materials and methods

### Study design, period and site

**Figure 1.**
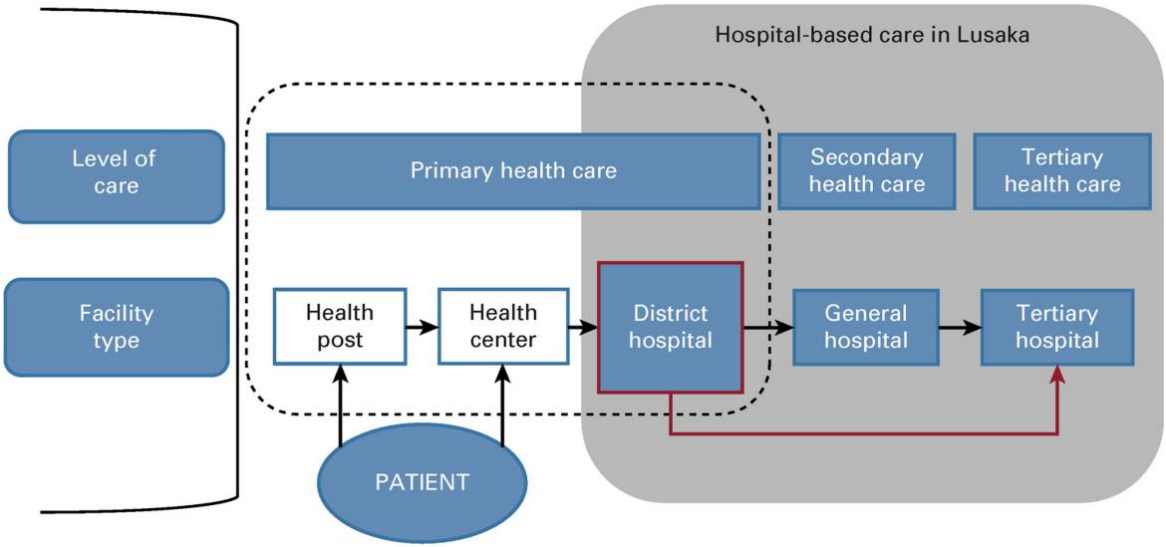
Structure of the healthcare and referral system in Zambia. Source: Songiso et al.^91^

A retrospective cross-sectional study was conducted from August 2023 to October 2023 using patients’ medical records (prescriptions and medical files) at St. Francis’ Mission Hospital located in the Katete district of Eastern Province in Zambia. Zambia is a low-income country located in southern Africa. The country’s economy is highly dependent on the mining and agriculture system, in addition to the construction and trade sectors.^89^ Zambia’s healthcare system is still developing and is made up of the public and private sectors. The primary healthcare system is composed of rural health posts (usually staffed by community health assistants, pharmacy personnel and nurses), health centres (usually staffed by clinical officers, pharmacy personnel, nurses and midwives) and district hospitals (staffed by specialists including paediatricians, general surgeons, obstetricians and gynaecologists, pharmacists, pharmacy technologists, nurses, physiotherapists, laboratory scientists, dentists, public health specialists, epidemiologists, infectious disease specialists, radiographers and internists).^90–92^ Regarding the referral system, the district hospitals make patient referrals to general and tertiary hospitals (Figure 1). St. Francis’ Hospital is a 350-bed capacity referral hospital providing medical and surgical services to the population in the Eastern Province of Zambia.^93^ This rural hospital was chosen for this study since inappropriate prescribing of antibiotics is more likely to occur in such hospitals, with ongoing ASP activities initially being concentrated in urban areas including Lusaka.^58^ In addition, this hospital accepts referrals from the rest of the Eastern Province (with a population of 1.5 million people) in Zambia, enhancing its importance for assessing current antibiotic prescribing patterns.^93,94^ Currently, 200 000 outpatients and inpatients access medical services at St. Francis’ Mission Hospital annually, from a previous population of 87 294 patients.^94,95^

### Sample size estimation and sampling criteria

In line with the WHO’s recommendations for investigating medicine use in health institutions, a minimum of 600 medical records should be included in a cross-sectional survey.^96,97^ In our study, 800 medical records were reviewed, thereby enhancing the robustness and representativeness of our findings. Medical records were selected using a systematic random sampling method from a pool of 21 600 prescriptions/medical files dating between 1 January 2023 and 30 June 2023. The 6 month study period was chosen to balance the need for a sufficiently large dataset with the feasibility of timely data collection and analysis post-COVID-19. To determine the sampling interval, we divided the total number of documents by the desired sample size, resulting in a sampling interval of 27. Consequently, every 27th medical record was selected for inclusion in the study. Patients’ medical records from other healthcare facilities were excluded from this study.

### Data collection

Data collection was conducted initially using the validated tool developed by the WHO (https://www.who.int/publications/i/item/who-dap-93.1) and used by others,^96,98,99^ building on earlier studies in Zambia.^65,79^ The tool collects reliable data on drug use in healthcare facilities and has been used extensively in previous studies.^4,47,60,100^ Information collected in this part of the study included records of the patient’s age and sex, diagnosis, the name of the antibiotic prescribed, the number of medicines prescribed by generic name, prescriptions with an antibiotic, and prescriptions with injection encounters. Additionally, we also reviewed whether the prescriptions for medicines were contained in the current Zambia Essential Medicines List (ZEML) and Standard Treatment Guideline (STG) and the indications for the prescribed antibiotics.^101,102^

Some of the indicators that were used in the study to assess the characteristics and the quality of prescribing included: (i) the average number of medicines per encounter; (ii) the percentage of medicines prescribed by their generic name (international non-proprietary name—INN); (iii) the percentage of encounters where an antibiotic was prescribed; (iv) the percentage of encounters where the medicine was given via an injection; and (v) the percentage of medicines prescribed from the EML or formulary.^60,96^ The rate of prescribing of generic (INN) versus branded medicines is important given anticipated savings.^103,104^ However, there can be concerns with the quality of generics among African countries, which needs addressing going forward.^105–107^ There are also concerns about high rates of prescribing of injectable antibiotics in hospitals.^108–110^ The prescribing of injectable antibiotics also causes possible harm to patients.^111^ If this prescribing behaviour continues, it will impact the length of hospital stay and associated costs. Therefore, ASPs in hospitals must be strengthened because they are often aimed at de-escalation where this is seen as a problem.^112,113^

The second part of the study involved a closer evaluation of the different antibiotics prescribed based on the AWaRe classification. The initial target is at least 60% of antibiotics prescribed should be from the ‘access’ list to help limit AMR.^6,33^ Some of the antibiotics that fall under the ‘access’ group include amoxicillin, amikacin, ampicillin, cloxacillin, clindamycin, metronidazole, tinidazole, benzylpenicillin, doxycycline, gentamicin, chloramphenicol, phenoxymethyl penicillin, cefalexin, sulfamethoxazole/trimethoprim (co-trimoxazole), benzathine penicillin, erythromycin, tetracycline and nitrofurantoin.^42,43^ Some examples of ‘watch’ group antibiotics include azithromycin, ceftriaxone, cefotaxime, cefepime, cefuroxime, imipenem/cilastatin, meropenem, ertapenem, ciprofloxacin, gatifloxacin, gemifloxacin, levofloxacin, streptomycin, tobramycin, vancomycin, oxytetracycline, kanamycin and neomycin.^42,43^ Furthermore, some examples of ‘reserve’ group antibiotics include aztreonam, daptomycin, fosfomycin, colistin, linezolid, minocycline and tigecycline.^42,43^

### Data management and analysis

Microsoft Excel (Microsoft, Redmond, WA, USA) was used to enter, sort and clean the data and thereafter analysed using Statistical Package for Social Sciences (SPSS) version 23.0 (IBM Corp., Armonk, NY, USA). Descriptive statistics were performed for demographic characteristics and other prescribing indicators and results were presented in tables and charts as frequencies and percentages.

### Ethics

Ethical approval was granted by the University of Zambia School of Health Sciences Research Ethics Committee (UNZASHREC) with a clearance ID of 202301270003. Permission to conduct data collection was obtained from the Senior Medical Superintendent (SMS) at St. Francis’ Mission Hospital. We observed the ethical issues of confidentiality to ensure that all the patient information collected was not accessed by other people. The data collected were stored on a password-protected computer and were only accessible to the research team.

## Results

### Sociodemographic characteristics of patients and medicine prescriptions

From a total of 800 prescriptions and medical files that were reviewed, the majority were for female patients (57.1%, *n* = 457), with most prescriptions for patients aged between 16 and 30 years (30.1%, *n* = 241) (Table 1).

**Table 1.** Sociodemographic characteristics of sampled medical records for patients at Saint Francis’ Hospital, Zambia: January 2023—June 2023

Variable	*n*	%
Gender		
* *Female	457	57.1
* *Male	343	42.9
Age category (years)		
* *0–15	210	26.2
* *16–30	241	30.1
* *31–45	167	20.9
* *>45	182	22.8

### Number of medicines prescribed per encounter

**Figure 2.**
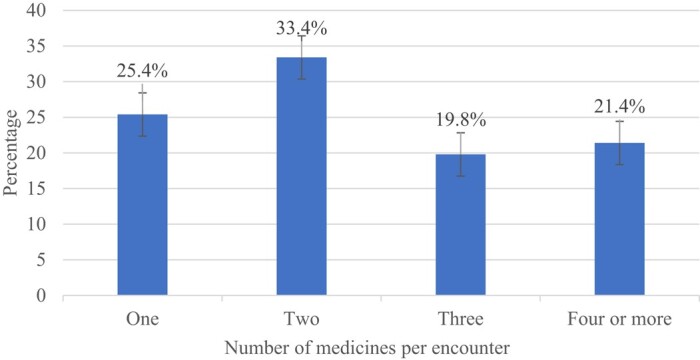
Number of medicines prescribed per encounter at Saint Francis’ Hospital, Zambia: January 2023—June 2023.

Of the 800 prescriptions that were included in the study, 33.4% (*n* = 267) indicated that most patients were prescribed two medicines, followed by 25.4% (*n* = 203) who were prescribed one medicine per encounter (Figure 2). Only 25.4% of the patients received a single medicine; this shows that most patients (74.6%) received two or more medicines, indicating a high practice of polypharmacy. There was an increased prescribing of medicines by injection at 28.4% (*n* = 227) of all medicines, which included antibiotics.

### Antibiotic prescribing patterns at Saint Francis’ Hospital

**Figure 3.**
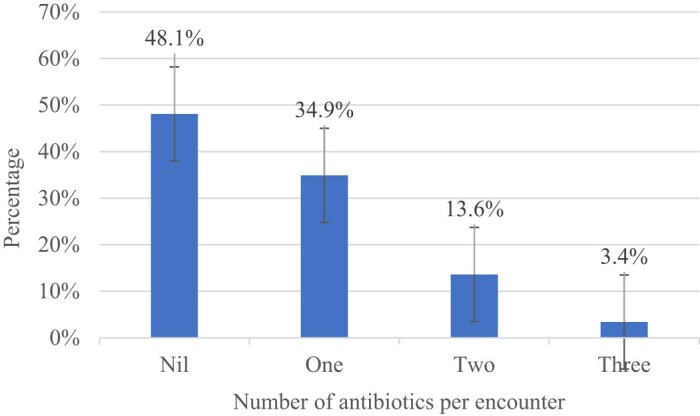
Number of antibiotics prescribed per encounter at Saint Francis’ Hospital, Zambia: January 2023—June 2023.

Of the 800 medical records reviewed, 51.9% (*n* = 415) were prescribed a total of 578 antibiotics, of which 34.9% (*n* = 279) had 1 antibiotic, 13.6% (*n* = 109) had 2 antibiotics, and 3.4% (*n* = 27) had 3 antibiotics prescribed (Figure 3). No patient had more than three antibiotics per encounter.

**Figure 4.**
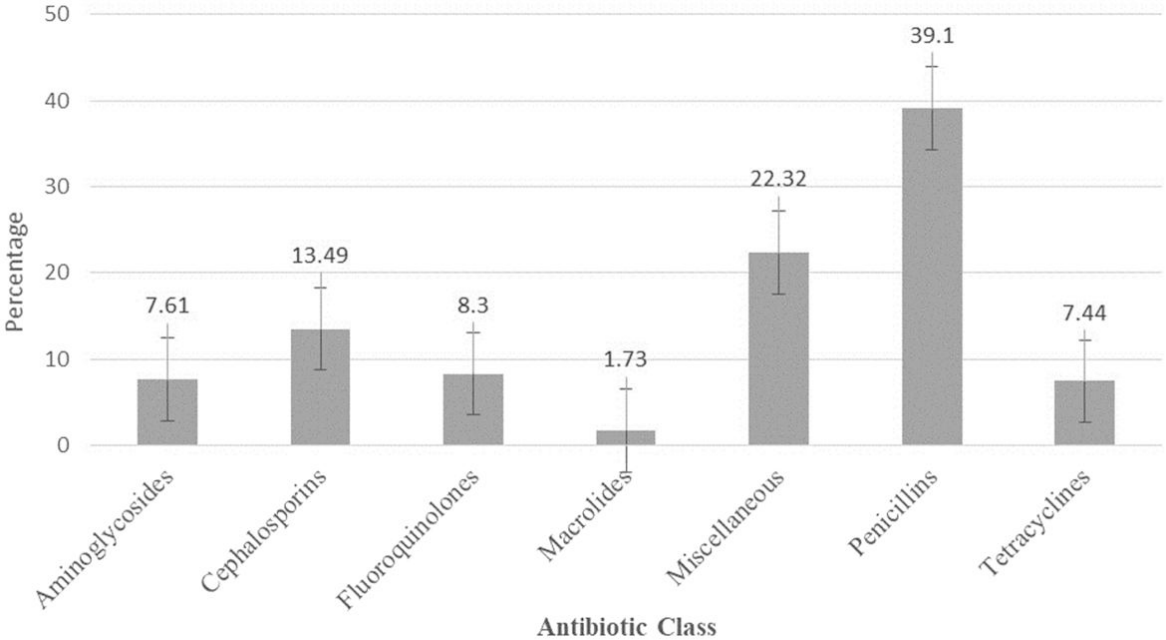
Percentage distribution of commonly prescribed antibiotics by class at Saint Francis’ Hospital, Zambia: January 2023—June 2023.

The penicillins accounted for the highest proportion of antibiotics at 39.1% of total encounters, with amoxicillin being the most frequently prescribed antibiotic (23.4%, i.e. 135 encounters) (Table 2, Figure 4). Metronidazole accounted for 17.1% of encounters, followed by ciprofloxacin (8%) with 46 encounters. Overall, among the antibiotics prescribed, the ‘access’ group constituted 77.1% and those from the ‘watch’ group 22.9% (Table 2). The results in Table 2 were presented based on the WHO AWaRe classification of antibiotics.^34,37^

**Table 2.** Distribution of commonly prescribed antibiotics by AWaRe classification at Saint Francis’ Hospital, Zambia: January 2023—June 2023

Commonly prescribed antibiotics	AWaRe classification	*n*	%
Amoxicillin	Access	135	23.4
Metronidazole	Access	99	17.1
Ciprofloxacin	Watch	46	8.0
Ceftriaxone	Watch	43	7.4
Benzylpenicillin	Access	42	7.3
Doxycycline	Access	40	6.9
Cefotaxime	Watch	24	4.2
Cloxacillin	Access	23	4.0
Gentamicin	Access	22	3.8
Neomycin	Watch	19	3.3
Chloramphenicol	Access	16	2.8
Phenoxymethyl penicillin	Access	16	2.8
Cefalexin	Access	14	2.4
Co-trimoxazole	Access	13	2.2
Benzathine penicillin	Access	11	1.9
Erythromycin	Access	10	1.7
Tetracycline	Access	3	0.5
Nitrofurantoin	Access	2	0.3

The most common conditions for which antibiotics were prescribed in this study included RTIs at 40.3% (24.9% for lower RTIs and 15.4% for URTIs) and sexually transmitted infections (STIs) at 9%. Antibiotics were also prescribed for skin and soft-tissue infections (7.6%), conjunctivitis (5.7%), gastrointestinal tract infections (5.2%) and urinary tract infections (UTIs) (5.2%). Antibiotics were also used less frequently for other diseases (Table 3). The high use of penicillin and cephalosporin antibiotics was for RTIs. Nitrofurantoin (0.3%) was rarely prescribed for patients, despite it being highly recommended for the treatment of UTIs in Zambia (Table 3).

**Table 3.** Distribution of common conditions treated with antibiotics at Saint Francis’ Hospital, Zambia: January 2023–June 2023

Diagnosis	*n*	%
Lower RTI	105	24.9
URTI	65	15.4
STI	38	9
Skin and soft-tissue infection	32	7.6
Conjunctivitis	24	5.7
Gastrointestinal tract infection	22	5.2
UTI	22	5.2
Obstetric post-Caesarean section prophylaxis	21	5
Sepsis	14	3.3
Prophylaxis in HIV patients	9	2.1
Febrile illness	7	1.7
Incomplete abortion	7	1.7
Pulpitis	6	1.4
Complete abortion	5	1.2
Tooth extraction	5	1.2
Rheumatic heart disease	4	1
Meningitis	4	1
Group 1 diseases	12	3
Group 2 diseases	19	3.8

Group 1 infections: septic abortion, post-Caesarean section for cephalopelvic disproportion, peritonitis, corneal abrasion, adult periodontitis and infective endocarditis. Group 2 infections: acute glomerulonephritis, cystic fibrosis, hepatorenal syndrome, malaria, jaundice secondary to cholecystitis, knee arthrotomy, lymphadenitis, lymphogranuloma venereum, pleural effusion, post-dental surgery, post-exploratory laparotomy, post-molar pregnancy extraction, post-operative cataract, proteus syndrome, pseudophakia, severe acute malnutrition with oedema, sickle cell disease, suspected lymphoma and umbilical hernia.

### Summary of WHO prescribing indicators of medicines at Saint Francis’ Hospital

The average number of medicines per encounter was 2.5 (2003 medicines prescribed from 800 patient files). Antibiotics were prescribed in 72.3% encounters, of which 28.4% were injectable. Furthermore, 96.5% of medicines were prescribed by generic name and 98% came from the ZEML (Table 4). The findings of the present study indicate that none of the studied indicators met the WHO prescribing indicators of medicines in hospitals (Table 4).

**Table 4.** Summary of WHO prescribing indicators of medicines at Saint Francis’ Hospital, Zambia: January 2023–June 2023

Prescribing indicators	Total number of medicines	Average (*n* or %)	WHO standard value
Medicines per encounter (*n*)	2003	2.5	1.6–1.8
Encounters with antibiotics (%)	578	72.3%	(20%–26.8%)
Encounters with injection (%)	227	28.4%	(13.4%–24.1%)
Medicines prescribed by generic name (%)	1933	96.5%	100%
Medicines from the ZEML (%)	1962	98%	100%

## Discussion

To the best of our knowledge, this is the first study that has evaluated the prescribing of antibiotics using the WHO prescribing indicators, across both ambulatory care and inpatient care in Zambia after the COVID-19 pandemic. The findings reveal a higher-than-average number of medicines being dispensed per patient encounter, with antibiotics constituting a notable 72.3% of the total encounters. This prevalence suggests a potentially alarming reliance on antibiotics, a concern that is amplified by the fact that penicillins, specifically amoxicillin, were the most commonly prescribed. Interestingly, a high proportion of medicines were prescribed by their generic name and sourced from the ZEML, signalling a high adherence to best practices in these specific areas.

The current study found that the average number of medicines prescribed per patient encounter was 2.5, which is higher than the range recommended by the WHO of 1.6–1.8. This finding is consistent with a previous study conducted in Lusaka, Zambia, which also reported an average of 2.5 medicines prescribed per encounter.^79^ In contrast to our findings, other studies have reported varying average numbers of medicines prescribed for a patient, with an average of 3.4 reported in Nigeria,^114^ 3.2 in Yemen^115^, 2.91 in India,^116^ 2.8 in Botswana^117^ and 2.09 in South Sudan.^118^ On the other hand, a lower average number of medicines prescribed per encounter was reported in other studies, with a 1.12 average reported in India,^119^ 1.14 in Cameroon,^120^ 1.6 in Ethiopia^4^ and 1.76 in Eritrea.^47^ The observed discrepancy in the number of prescribed medications per patient encounter may be attributed to differences in the research environments and the specific prescribing practices employed across various medical specialities.^121,122^ We also assume that the number of medications prescribed for a patient may be informed by patient comorbidities and disease severity, among others.

Furthermore, the present study found that antibiotics were prescribed for 51.9% of the medical records, which translated into 72.3% of total antibiotic encounters. This antibiotic encounter surpassed the WHO-recommended range of 20%–26.8%. Intriguingly, this finding is similar to findings from previous studies, with 53% having antibiotics prescribed in Eritrea,^47^ 52.3% in Ethiopia^46^ and 52.4% in Pakistan.^123^ Our findings are higher than the combined findings across the WHO African region of 46.8% of patient encounters in which antibiotics were prescribed^44^ and 42.7% in Botswana.^117^ Our findings were lower than the 98.4% to 100% of encounters where antibiotics were prescribed in Niger and Uganda,^124^ 85% in Jordan,^125^ 84.2% in Yemen,^115^ 81.3% in Nigeria^114^ and 78.8% in Ghana.^126^ The factors contributing to the excessive use of antibiotics might include health facilities relying on empirical treatment instead of confirmed diagnosis. Furthermore, the lack of essential diagnostic tools to differentiate between bacterial and viral infections, especially in LMICs, is a contributing factor to the inappropriate prescribing of antibiotics in healthcare facilities.^127^ The present study also found that 13.6% of patients were prescribed two antibiotics, and 3.4% (*n* = 27) had three antibiotics prescribed. This also needs to be monitored, especially if there was no culture and sensitivity testing undertaken to guide the antibiotic choice. Such prescribing behaviour will increase AMR if not addressed, and impact Zambia achieving the goals within its NAP.^87^

Our study found that penicillins were the predominant class of antibiotics prescribed, and these are typically from the ‘access’ group, followed by cephalosporins, and fluoroquinolones, similar to an earlier study in Zambia and Jordan, where penicillins were the most widely prescribed antibiotics,^65,128^ with studies in Botswana, Cameroon, Ghana and Tanzania also showing high rates of prescribing of amoxicillin.^86,99,117,120^ This was followed by metronidazole and ciprofloxacin, again similar to Botswana, Cameroon, Ghana and Tanzania.^86,99,117,120^ This may reflect comparatively high rates of STIs as well as UTIs, in addition to RTIs, in these countries, with high rates of RTIs also seen in other countries.^99,116,128,129^ Respiratory infections are prevalent in Southern Africa, including Zambia, due to the abundance of infectious agents (bacteria, viruses and fungi) and limited healthcare and sanitation access, creating conditions that favour pathogen transmission. Overcrowded living conditions could also worsen the spread of respiratory infections in the region.^130,131^

The present study findings are different from those in Pakistan, where in one study cephalosporins (81.5%) were the most commonly prescribed antibiotic class, followed by penicillins (6.4%) and fluoroquinolones,^123^ and in Palestine,^132^ where ceftriaxone was the most commonly prescribed antibiotic, followed by cefazolin. A similar study in India reported that metronidazole and ceftriaxone were highly prescribed.^133^ There are also other reports of high rates of prescribing of ceftriaxone in several studies, including those in hospitals in Zambia during the COVID-19 pandemic, enhanced by ceftriaxone being included in treatment guidelines for the management of COVID-19 among African countries.^39,64,74,77,134,135^ This though was not seen in our study (Table 2), with overall 77.1% of antibiotics encouragingly prescribed from the ‘access’ group and 22.9% from the ‘watch’ group. This finding in our study is higher than an initial target of 60% from the ‘access’ group.^6,33^ A study conducted in Ghana also found high use of the ‘access’ group antibiotics.^126,136,137^ Other studies have reported deviations from the WHO AWaRe classification recommendations and reported high use of ‘watch’ antibiotics in hospitals.^138–141^

The present study found that antibiotics were prescribed mostly for RTIs, STIs and skin and soft-tissue infections. This outcome aligns with findings from other studies, where RTIs were the most common conditions in which antibiotics were often prescribed.^99,116,128,129^ A similar study conducted in Lusaka, Zambia also reported that most antibiotics were prescribed for RTIs.^74^

This study found that 28.4% of the prescribed antibiotics were injections, which is significantly higher than the range that the WHO recommends, which is 13.4%–24.1%. This may reflect the fact that our study included both inpatients and ambulatory care patients. This though needs further investigation, especially if there is prolonged use of injections in hospitals without de-escalating to oral antibiotics to hasten discharge. Our figures were higher than the 17.5% of antibiotics given by injection in paediatric outpatient departments in Nigeria,^114^ 6.3% in outpatients in Ethiopia,^4^ 7.8% among community pharmacies in Eritrea^47^ and 11.8% in a previous study in a University Teaching hospital in Zambia.^79^ However, they were significantly lower than the 84.8% seen among inpatients in Ethiopia.^46^ Consequently, the location of care is important when assessing whether there is a high use of injections or not.

Intriguingly, the current study found that 96.5% of antibiotics were prescribed by their generic names, which was slightly lower than the 100% threshold standard recommendation by the WHO. This result was similar to the rate that was reported in Ethiopia (96%).^4^ However, in our findings the percentage prescribing antibiotics by generic names was higher than that observed in other studies, including 84.4% in Tanzania,^99^ 78.6% in Botswana,^117^ 55% in Palestine,^132^ 51.6% in Nigeria,^114^ 41% in Yemen^115^ and 10.05% in India.^116^ In contrast, the results of the current study indicated a slightly lower rate of prescribing antibiotics by their generic names compared to 97.6% and 98.83% reported in two different studies in Ethiopia^46,47^ and 98.36% in Cameroon.^120^ Using brand names for medicines can have various consequences, including the potential for medication errors due to the similarity and resemblance of some brand names.

Our study also revealed that 98% of the medicines prescribed came from the current ZEML, which is close to the 100% that is recommended by the WHO. This finding was comparable to those found in earlier research, in which the percentage of medicines given from the national EML was 99.87% in Cameroon,^120^ 98.1% in Zambia,^79^ 98% from the Gaza Strip in Palestine,^132^ 97.6% in Tanzania^99^ and 97.5% in Ethiopia.^100^ However, the rate of prescribing antibiotics from the national EML that was found in our study exceeded the results seen in other studies that were similar to it. For example, the prescribing of medicines from the national EML was reported to be 96.1% in Botswana,^117^ 84.8% in India^116^ and 44% in Yemen.^115^ Intriguingly, research carried out in Ethiopia and Jordan discovered that all (100%) of the antibiotics prescribed were from the respective national EMLs. This finding is in line with the guidelines made by the WHO and suggests that the EML was followed to its fullest extent.^46,129^

Overall, the study has highlighted potential areas for improvement that can be part of future ASPs, with ASPs now being routinely instigated across Africa to improve future prescribing. These include the necessity for giving antibiotics by injection, which for inpatients can be extended to assess whether there are delays in de-escalating to oral antibiotics. In addition, the need for routinely documenting the rationale for prescribing antibiotics in the first place across the sectors, especially if more than one antibiotic is being prescribed to patients. Lastly, there is a need to routinely assess the quality of prescribing antibiotics based on the recently available AWaRe book in line with suggestions for other African countries.^32,33,142,143^ For instance, nitrofurantoin (0.3%) was rarely prescribed for patients with UTIs, despite it being highly recommended for the treatment of UTIs in Zambia and as an alternative antibiotic in the AWaRe book. In the first instance, this can include prescribing targets for percentage adherence to current guidance, with these targets regularly monitored and discussed.^142,143^

The results of this study highlighted the importance of designing educational interventional activities specifically geared towards the prudent prescription of antibiotics. Educational interventions and positive behavioural change regarding the prescribing of antibiotics are critical in addressing AMR.^53,144–150^ In addition, our findings point to the necessity of developing and putting into practice measures that encourage the rational prescribing and use of antibiotics, which is consistent with the findings of other research.^1,24,25,151–153^ In addition, there is a need to promote hospital-based ASPs to assist prescribers in adhering to treatment guidelines and prescribing antibiotics for the appropriate patient, at the appropriate time, in the appropriate dose, for the appropriate duration, via the appropriate route of administration, and for the appropriate indication, as was reported in other studies.^60,154–160^

We are aware that there are some limitations to our research. Firstly, we only undertook this post-COVID-19 study in one hospital. Secondly, there are always limitations with retrospective studies depending on the details contained within patients’ medical files. Despite these limitations, we believe the findings are noteworthy and provide important insights that can guide potential changes in policy and practice at this facility and others in Zambia to achieve the AMR goals within the Zambian NAP.

### Conclusions

This study found a higher than average number of medicines prescribed per patient encounter. Additionally, antibiotics were prescribed in 72.3% of encounters, of which 28.4% were injectable. The most frequently prescribed antibiotics were amoxicillin (23.4%—access), metronidazole (17.1%—access), ciprofloxacin (8%—watch) and ceftriaxone (7.4%—watch), with 77.1% overall from the ‘access’ list. Slight deviations were observed in the prescribing of antibiotics by generic names and from the national EML. Therefore, potential targets for ASPs in this hospital include assessing current prescribing against agreed guidance in the AWaRe book, reducing the need for antibiotics to be given by injection where pertinent, and reducing the extent of multiple antibiotics given to patients without assessing the need through culture and sensitivity testing. There is also a need to follow this up in future studies with the implications for reducing AMR in Zambia in line with the goals of the NAP.

## Supplementary Material

dlae023_Supplementary_Data

## Supplementary data

The data collection tool (prescribing indicator form) is available as Supplementary data at *JAC-AMR* Online.
