# Supplementary material for: Hospital prescribing patterns of antibiotics in Zambia using the WHO prescribing indicators post-COVID-19 pandemic: findings and implications
Source: JAC Antimicrob Resist. 2024 Feb 22;6(1):dlae023. doi: 10.1093/jacamr/dlae023 (PMC10883698; doi:10.1093/jacamr/dlae023)
Supplement: dlae023_Supplementary_Data [file dlae023_supplementary_data.docx]

**Hospital Prescribing Patterns of Antibiotics in Zambia using the WHO Prescribing Indicators Post-COVID-19 Pandemic: Findings and Implications**

**Data collection tool**

| **No** | **Type(R/P)** | **Date of Rx** | **Age (yrs)** | **Sex** | **#**  **Medicines** | **#**  **Generic** | **Antibiotic (0/1)** | **Name of Antibiotic** | **Injections (0/1)** | **# on EML** | **Diagnosis*** |
| --- | --- | --- | --- | --- | --- | --- | --- | --- | --- | --- | --- |
| 1 |  |  |  |  |  |  |  |  |  |  |  |
| 2 |  |  |  |  |  |  |  |  |  |  |  |
| 3 |  |  |  |  |  |  |  |  |  |  |  |
| 4 |  |  |  |  |  |  |  |  |  |  |  |
| 5 |  |  |  |  |  |  |  |  |  |  |  |
| 6 |  |  |  |  |  |  |  |  |  |  |  |
| 7 |  |  |  |  |  |  |  |  |  |  |  |
| 8 |  |  |  |  |  |  |  |  |  |  |  |
| 9 |  |  |  |  |  |  |  |  |  |  |  |
| 10 |  |  |  |  |  |  |  |  |  |  |  |
| 11 |  |  |  |  |  |  |  |  |  |  |  |
| 12 |  |  |  |  |  |  |  |  |  |  |  |
| 13 |  |  |  |  |  |  |  |  |  |  |  |
| 14 |  |  |  |  |  |  |  |  |  |  |  |
| 15 |  |  |  |  |  |  |  |  |  |  |  |
| 16 |  |  |  |  |  |  |  |  |  |  |  |
| 17 |  |  |  |  |  |  |  |  |  |  |  |
| 18 |  |  |  |  |  |  |  |  |  |  |  |
| 19 |  |  |  |  |  |  |  |  |  |  |  |
| 20 |  |  |  |  |  |  |  |  |  |  |  |
| 21 |  |  |  |  |  |  |  |  |  |  |  |
| 22 |  |  |  |  |  |  |  |  |  |  |  |
| 23 |  |  |  |  |  |  |  |  |  |  |  |
| 24 |  |  |  |  |  |  |  |  |  |  |  |
| **Total** | | |  |  |  |  |  |  |  |  |  |
| **Average** | | |  |  |  |  |  |  |  |  |  |
| **Percentage** | | |  |  | **% of total medicines** | **% of total cases** | **% of total cases** |  | **% of total medicines** |  |  |
